# Gut Microbiota Composition in Relation to the Metabolism of Oral Administrated Resveratrol

**DOI:** 10.3390/nu14051013

**Published:** 2022-02-28

**Authors:** Mingfei Yao, Yiqiu Fei, Shuobo Zhang, Bo Qiu, Lian Zhu, Fang Li, Björn Berglund, Hang Xiao, Lanjuan Li

**Affiliations:** 1 State Key Laboratory for Diagnosis and Treatment of Infectious Diseases, National Clinical Research Center for Infectious Diseases, Collaborative Innovation Center for Diagnosis and Treatment of Infectious Diseases, The First Affiliated Hospital, School of Medicine, Zhejiang University, Hangzhou 310003, China; mingfei@zju.edu.cn (M.Y.); 22018095@zju.edu.cn (Y.F.); zhangshuobo197111@163.com (S.Z.); 22118248@zju.edu.cn (B.Q.); 2 School of Basic Medical Sciences and Forensic Medicine, Hangzhou Medical College, Hangzhou 310053, China; 2020000043@hmc.edu.cn; 3 Cardiometabolic Genomics Program, Division of Cardiology, Department of Medicine, Columbia University Irving Medical Center, 630 W 168th St., P&S10-401, New York, NY 10032, USA; fl2532@cumc.columbia.edu; 4 Department of Biomedical and Clinical Sciences, Linköping University, SE-58183 Linköping, Sweden; bjorn.berglund@liu.se; 5 Department of Food Science, University of Massachusetts, Amherst, MA 01003, USA; 6 Jinan Microecological Biomedicine Shandong Laboratory, Jinan 250021, China

**Keywords:** resveratrol (RSV), metabolites, gut microbiota, mouse model, *Ligilactobacillus salivarious* Li01

## Abstract

Resveratrol (RSV) has been confirmed to confer multiple health benefits, and the majority of RSV tends to be metabolized in the gut microbiota after oral administration. In this study, the metabolism of RSV was investigated by using mouse models with distinct gut microbiota compositions: germ-free mice colonized with probiotics, conventional mouse, and DSS-induced colitis mouse models. The results demonstrated that in feces, the metabolites of RSV, including resveratrol sulfate (RES-sulfate), resveratrol glucuronide (RES-glucuronide), and dihydroresveratrol, significantly increased after probiotics colonized in germ-free mice. Furthermore, RES-sulfate and RES-glucuronide were below the detectable limit in the feces of conventional mice, with dihydroresveratrol being the dominant metabolite. Compared to the conventional mice, the ratio of *Firmicutes*/*Bacteroides* and the abundance of *Lactobacillus* genera were found significantly elevated in colitis mice after long-term ingestion of RSV, which shifted the metabolism of RSV in return. Our study provided critical implications in further application of RSV in foods and food supplements.

## 1. Introduction

3,5,4′-trihydroxy-trans-stilbene, or resveratrol (RSV), a natural stilbenoid polyphenol, is present abundantly in grapes, berries, peanuts, and some food products. The compound is composed of two phenolic rings linked together by a double styrene bond, and has both a cis- and a trans- isomer [1]. RSV has attracted extensive interest owning to its health benefits, which include preventing and slowing the progression of a wide variety of chronic diseases, such as cardiovascular disease [2], diabetes [3], cancer [4], and inflammatory bowel disease [5]. However, despite the large quantity of published studies on RSV, the physiologic effects of RSV and the concrete mechanisms by which they work are not yet clearly understood.

Poor water solubility and extensive metabolism lead to low bioavailability of RSV [6]. After oral administration, RSV is metabolized by phase II enzymes and form sulfate and glucuronide metabolites in the gastrointestinal tract and the liver, with trans-resveratrol-3-O-glucuronide and trans-resveratrol-3-sulfate the most abundant metabolites of RSV [7,8]. Metabolites exert more favorable health benefits. For example, resveratrol sulfate (RES-sulfate) was found to provide an intracellular reservoir for generation of parent RSV and could enhance the anticancer effects [9]. The major metabolite of RSV produced by gut microbiota is dihydroresveratrol (DHR) [10], which has been demonstrated to have more effective antioxidant than some vitamins, such as vitamin E.

RSV mediated changes in the gut microbiome may be an important mechanism by which RSV mediates its beneficial renal function effects [11]. Hence, it is essential to understand the extent to which RSV is metabolized. The metabolism of RSV in gut microbiota shows interindividual differences, which is highly dependent on the gut microbiota composition [12]. Whereas the composition of the gut microbiota can influence the metabolism of RSV, RSV and its metabolites can also alter diversity and composition of gut microbiota. Hence, it has been hypothesized that understanding these bidirectional interactions between RSV and gut microbiota is the key to revealing the mechanisms of RSV efficacy. In order to characterize these interactions, this study investigated the effect of different types of gut microbiota, in different types of mouse models, on the metabolism of orally administrated RSV. The mouse models included germ-free mice colonized with *Ligilactobacillus salivarious* Li01 (CGMCC7045, Li01), conventional mouse (Specific pathogen free), and dextran sulfate sodium (DSS)-induced colitis mouse models. Li01 is probiotic isolated from healthy individual feces and exhibited health benefits [13]. Furthermore, we used metagenomic analyses to identify if certain effects on the metabolism of RSV could be linked to the relative abundance of specific bacterial taxa in the gut microbiota.

## 2. Materials and Methods

### 2.1. Materials

*Lactobacilli* MRS agar and *Lactobacilli* Demann, Regosa, Sharpe (MRS) broth were purchased from Oxoid (Oxoid, Basingstoke, Hampshire, UK). RSV and its metabolites RSV sulfate, RSV glucuronide, and DHR standards were obtained from Aladdin Co. in Shanghai, China. Methanol, acetonitrile, and all other compounds were obtained from Sinopharm Chemical Reagent Co., Ltd.in Shanghai, China. QIAamp Fast DNA Stool Mini Kit (Qiagen, Hilden, Germany) was used to extract bacterial DNA. Deionized water used in all experiments was obtained from a commercial water purification system (Nanopurediamond, Thermo Scientific, Waltham, MA, USA).

### 2.2. Bacterial Propagation

Li01 was stored routinely in MRS broth containing 50% glycerol at −80 °C. For bacterial propagation, 0.1 mL bacterial solution was inoculated in 50 mL MRS broth and was incubated at 37 °C for 24 h in anaerobic chamber (Electrotek, West Yorkshire, UK). After 24 h incubation, bacterial MRS solution was centrifuged at 1800× *g* for 5 min (Eppendorf, Germany). Then, the supernatant was discarded and the pellet was washed with saline buffer twice. The probiotics were resuspended with 1 mL saline buffer to make a bacterial suspension of 10^9^ CFU/mL for use as gavage for germ-free mice.

### 2.3. Establishment of Bacterial Colonized Germ-Free Mouse Model

Twelve six-week-old male germ-free ICR mice were randomly divided into two groups (*n* = 6) and kept at separated sterile isolators (Suzhou Fengshi Laboratory Animal Equipment Co., Ltd., Suzhou, China), according to the methods established before [14]. For RSV group, mice were provided with 0.2 mL PBS on the first day and fed with AIN93G diet (200 g of casein and 70 g of soybean oil/kg diet) [15] for 7 days. On the 8th day, the diet was changed into AIN93G supplemented with 500 ppm RSV (Changzhou SYSE Bio-tec Co., Ltd., Changzhou, China, RSV diet) and fed for another 7 days. Mice were sacrificed on the last day of the experiment. Feces, urine, plasma, and tissues were collected and stored in −80 °C freezer for further analysis. For RSV + Li01 group, mice were fed with 0.2 mL Li01 solution (~10^9^ CFU/mL) on the first day of the experiment and fed with AIN93G diet for 7 days until the Li01 probiotic cells were colonized and remained stable. From the 8th day, mice were fed with RSV diet for another 7 days. Feces were collected on the 4th, 7th and 12th days to determine the concentration of bacteria using a plate count method. On the last day of the experiment, they were sacrificed and tissue samples were collected for further analysis. Mouse food was weighed before and after the experiments and the ingested amount was quantified.

### 2.4. RSV Metabolism in Conventional Mice

Ten 5-week-old specific pathogen-free (SPF) C57BL/6 male mice were purchased from Zhejiang Laboratory Animal Center (Hangzhou, China) and maintained in pathogen-free conditions. Mice were fed with 0.2 mL PBS on the first day and provided with normal diet for 7 days. From the 8th day, each mouse was taken 30 mg RSV by intragastric gavage for continuous 7 days. On the 14th day, all mice were sacrificed. Feces, urine, serum, colonic content, liver, and kidney were collected and stored in −80 °C freezer for further analysis.

### 2.5. RSV Metabolism in DSS-Induced Colitis Model

Thirty 5-week-old SPF C57/BL6 mice were randomly divided into three groups (*n* = 10) including control group (conventional healthy mice), NS group (DSS induced colitis mice) and RSV group (DSS induced colitis mice with RSV treatment) and maintained in pathogen-free conditions. Each mouse was labeled and their weight was recorded daily. Except the mice in control group, the other two groups were fed with drinking water with 3% DSS at the first week and third week, in order to induce colitis. At the second and fourth week, 200 μL saline buffer or solution containing 30 mg RSV was fed to the mice every day in NS and RSV groups by oral gavage.

Feces and urine of all mice were collected at 14 and 29 days. All the mice were sacrificed on the 29th day and the serum, colonic feces, and entire organs including liver and kidney were collected for LC-MS/MS analysis or metagenome sequencing.

### 2.6. 16SrDNA Genes-Based Metagenomics

Bacterial DNA was extracted from colonic fecal samples using QIAamp Fast DNA Stool Mini Kit (Qiagen, Hilden, Germany). The V3-V4 variable regions of bacterial 16S rDNA genes were amplified with forward primer 5′-CCTACGGGNGGCWGCAG-3′and reverse primer 5′-GACTACHVGGGTATCTAATCC-3′ by using a previously described protocol [16]. The PCR products were purified by using AMPure XT beads (Beckman Coulter Genomics, Danvers, MA, USA) and quantified by using Qubit (Invitrogen, Waltham, MA, USA). Pooled amplicons were sequenced on an Illumina MiSeq platform. UPARSE was used to conduct quality control on raw data. Each sequence was classified to perform taxonomy-community analysis by using the Microbial Ecology Ribosomal Data base Project (RDP) database (http://rdp.cme.msu.edu/, accessed on 14 August 2020).

### 2.7. Extraction and LC-MS/MS Analysis of RSV and Its Metabolites

The extraction process of RSV and its metabolites from feces, serum, and tissue samples was operated under room temperature. Then, 20 μL of each sample was mixed with 50 μL tolbutamide as internal standard, 20 μL matrix as well as 200 μL acetonitrile. After vortex for 10 min, 200 μL supernatant was taken and diluted with 200 μL acetonitrile: water (*v*/*v*, 50/50) solution before analysis by an AB Sciex LC-MS system (5500). The recovery of RSV extraction, in this study, was above 90% for all samples.

RSV and its metabolites were then quantified and analyzed using Analyst1.6.3 (Sciex) and Watson Lims 7.5 (Thermo). An LTQ-Orbitrap mass spectrometer (Thermo Scientific, Bremen, Germany) to was employed to perform mass spectral analysis and the ESI was set to negative ion mode. The separation was implemented by Venvsil MP C18 column (2.1 mm × 100 mm, 3.5 μm). The mobile phase composed of (A) MNH_4_OAC/FA (100/5, *v*/*v*) and (B) acetonitrile. The optimized gradient was: 0–0.4 min, 45% B; 1.0 min, 1.0–3.0 min, 85% B; and 3.2 min, 45% B. The flow rate was set at 0.6 mL/min with the column temperature at 40 °C, and the injection volume of each sample was 15 μL.

**Figure 1:**
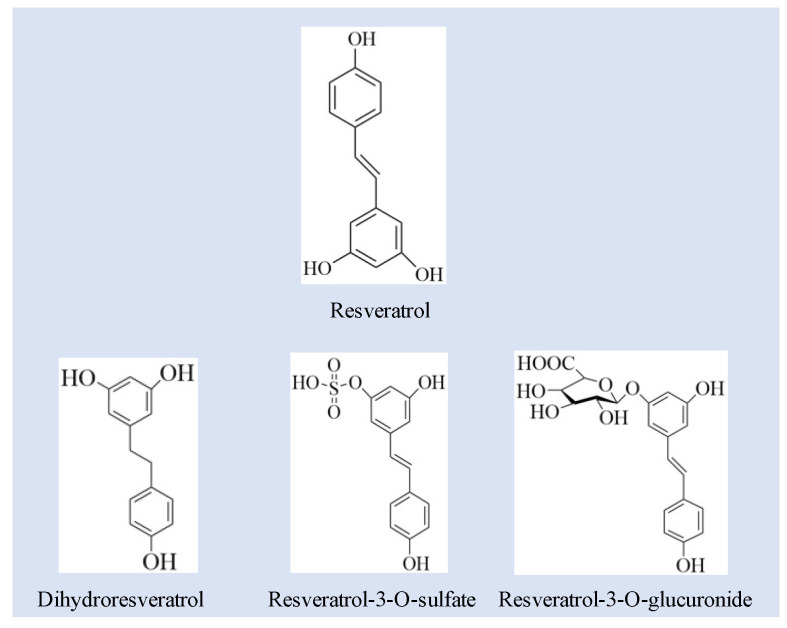
The structures of resveratrol and related metabolites including dihydroresveratrol, resveratrol-3-O-sulfate, resveratrol-3-glucuronide.

The MS detector was set to the multiple reaction monitoring (MRM) mode using the quantification ions *m*/*z* 227 ([M-H]^−^) → 185 (loss of C_2_H_2_O)); 403 ([M-H]^−^) → 227 [M-H]^−^); 307 ([M-H]^−^) → 185 ([M-H]^−^); and 229 ([M-H]^−^) → 123 ([M-H]^−^) for RSV, RES-glucuronide, RES- sulfate, and DHR, respectively (Figure 1). The lowest limit of quantification was 5 ng/mL in tissues and feces and 1 ng/mL in the serum.

### 2.8. Data Analysis

Statistical analyses were performed by GraphPad Prism (V.9.2.0). Independent-samples *t*-test and one-way ANOVA with LSD’s post hoc test were used to test median differences in of RSV and RSV metabolites. For nonparametric test, Mann–Whitney U test (two groups) or Kruskal–Wallis H test (multiple groups) was used to test mean differences.

## 3. Results and Discussion

### 3.1. Quantification of RSV and RSV Metabolites

The metabolism of RSV in the gastrointestinal tract has been widely investigated by in vitro and in vivo studies. The major metabolites of RSV have been identified to be phase II metabolites: glucuronide and sulfate conjugates, and no phase I metabolites formed when tested on a Caco-2 cell model [17]. DHR as a phase I metabolite of RSV, is mainly formed by the hydrogeneration of the double bond with the help of microflora [7]. Figure 1 exhibited the chemical structures of RSV and its metabolites related with this study. As has been reported previously, RES-sulphate, RES -glucuronide and DHR are the main metabolites of RSV (Figure 1) [18,19,20,21].

The detailed MS/MS chromatograms of RSV and metabolites were shown in Table 1. The molecular ion of RSV was at *m*/*z* of 227.0702 (C_14_H_11_O_3_). For RES-sulfate, the [M-H]- ions produces at *m*/*z* 307.0281 (C_14_H_11_O_6_S). The ion corresponding to RSV (*m*/*z* 227) through the neutral loss of the sulfate unit (*m/z* 80) from the RES-sulfate, and then the *m*/*z* 227 was fragmented to *m*/*z* 185 for the further loss of 42 amu (C_2_H_2_O) from RSV. Molecular ions of RES-glucuronide formed at *m/z* of 403.1029 (C_20_H_19_O_9_), and the MS fragment indicated that a glucuronide (176 Da) added to the RSV (227 Da). The deprotonated molecular ion of DHR arose at *m/z* 229.0865 (C_14_H_13_O_3_), and the fragment ion [M-H-H_2_]^−^ arose at *m/z* 277 [22].

### 3.2. Gut Microbiota Composition in Different Mouse Models

**Figure 2:**
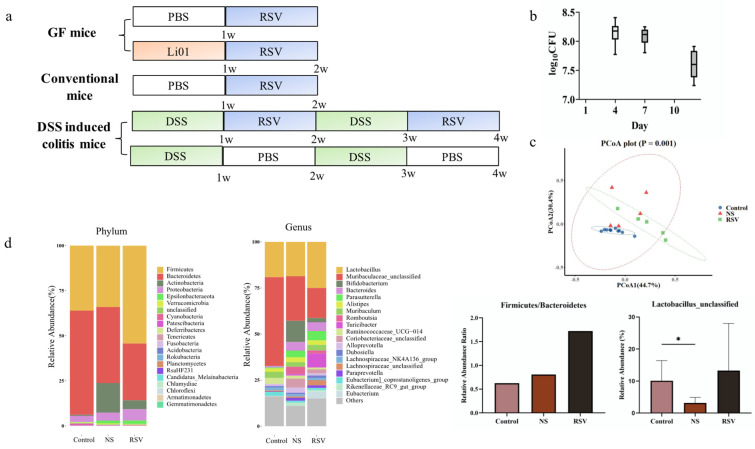
Gut microbiota composition in different mouse models. (**a**) Flow chart of animal studies in germ-free mice, conventional, and DSS-induced colitis mice. (**b**) Impact of RSV on colonized Li01 in germ-free mice. (**c**) Beta diversity evaluated by principal coordinates analysis (PCoA) of weighted unifrac distance. PCoA1 and PCoA2 represent the top two principal coordinates that captured most of the diversity. The fraction of diversity captured by the coordinate is given as a percentage. Groups were compared using analysis of similarities (ANOSIM) method. (**d**) Gut microbiota composition in healthy mice and DSS-induced colitis mice (before and after RSV intervention) analyzed by 16S rRNA gene sequencing analysis. Data are presented as mean ± SD. Significant difference was represented by * *p*  <  0.05, and analyzed by a one-way ANOVA with LSD’s post hoc test.

Gut microbiota shows pronounced interindividual differences, these differences may greatly affect the production of RSV metabolites. In this study, we established three mouse models (Figure 2a) in which the gut microbiota greatly differed. The first model consisted of germ-free mice which had been colonized by gavage with a bacterial strain *Ligilactobacillus salivarious* Li01 (Li01). Fecal quantities of were observed as shown in Figure 2b. The bacteria in stool samples were measured by a plate counting method and the results show the concentration of bacteria ranges from 7.6 to 8.2 log CFU/g, indicating bacteria was successfully colonized and a stable count of Li01 was maintained.

Conventional mice and DSS-induced colitis mice were used as other models. Figure 2c, d show the gut microbiota composition of DSS-induced colitis mice (NS group) and healthy conventional mice (control group) based on 16S rRNA gene sequencing. The diversity of gut microbiota was determined by principal coordinates analysis (PCoA) of weighted unifrac distance. As shown in Figure 2c, gut microbiome structure was significantly different among control, NS and RSV groups (ANOSIM, *p* = 0.001). Besides, the RSV group represents the gut microbiota composition after intervention with RSV. At the phylum level, the mice in the NS group and RSV had higher abundance of *Epsilonbacteraeota*, *Actinobacteria* (Kruskal–Wallis test, *p* value = 0.002, 0.03) and lower abundance of *Bacteroidetes* (*p* = 0.03) compared with mice in the control group. Compared to control and NS group, the ratio of *Firmicute/Bacteroidetes* significantly increased in RSV group, indicating a recovery of gut microbiota. At the genus level, among the 20 most prominent gut microbe species, RSV group had higher abundance of *Bifidobacterium* and *Rikenellaceae*_RC9_gut_group (Kruskal–Wallis test, *p* value = 0.003, 0.0069) and lower abundance of *Muribaculaceae* (Kruskal–Wallis test, *p* value = 0.0014). Obviously, although not statistically significant, the abundance of *Lactobacillus* was higher in RSV group mice compared to mice in the DSS group and the control group, which was consistent with previous studies in which RSV treatment improved the relative abundance of *Lactobacillus* in colitis mouse models [23]. Additionally, one of the *Lactobacillus* species were found higher in control and RSV group than in NS group.

### 3.3. RSV Metabolism in Germ-Free Mouse Model

Germ-free mice are widely used for studying the role of the gut microbiota on gastrointestinal physiology. The metabolic capacity will be significantly influenced when the gut microbiota is missing or in a state of dysbiosis. In this section, the metabolism and distribution of RSV in germ-free mice was investigated

**Figure 3:**
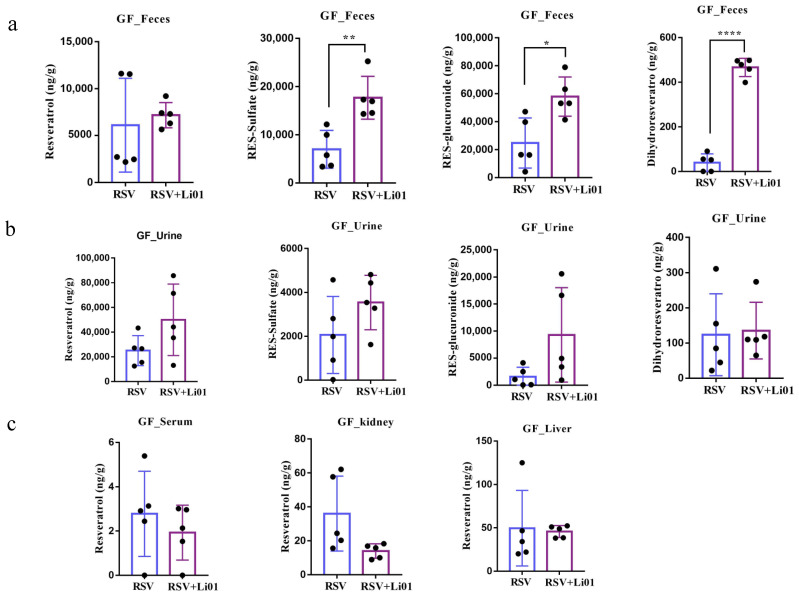
Quantification of RSV and its metabolites in feces (**a**), urine (**b**), serum, and tissues (**c**) in germ-free mice with/without Li01 colonization. Data are presented as mean ± SEM (*n* = 5). Significant difference was represented by * *p*  <  0.05, ** *p*  <  0.01, **** *p*  <  0.0001, and analyzed by either independent-samples *t*-test or nonparametric test (Mann–Whitney U-test).

Dietary RSV was fed to the germ-free mice colonized with or without Li01. As shown in Figure 3a, the levels of fecal RSV were not statistically different between mice in the two groups. However, RSV metabolites, including RES-sulfate (*p* = 0.009), RES-glucuronide (*p* = 0.03) and DHR (*p* = 0.02), were significantly increased in mice colonized with Li01. Among them, RES-sulfate and RES-glucuronide are the main metabolites of RSV and their concentration were observed to be around hundreds of folds compared to DHR., since RES-sulfate, RES-glucuronide is mainly formed in the enterocytes and no gut microbiota present in the large intestine of germ-free mice. However, DHR increased more than a hundred-fold after Li01 was colonized. DHR has been confirmed to be a metabolite formed in the intestine by the hydrogenation of the double bond of RSV by gut microbiota [24]. The above results indicated that the probiotic Li01 assisted with the metabolism of RSV into DHR.

The excretion of RSV and metabolites were also determined in urine sample. Although not statistically significant (*p* = 0.163, *p* = 0.119) (Figure 3b), the level of RES-sulfate and RES-glucuronide increased in the Li01 colonized group, which may be associated with increased metabolism of RSV in the gut microbiota. The level of RSV was also slightly elevated while DHR was similar in two groups.

Figure 3c shows the distribution of RSV in plasma and tissues. The concentration of RSV was very low in serum, only 2~6 ng/g. Although the accumulation of RSV was much higher in liver and kidney (20~100 ng/g) compared to that in serum, the ratio can be predicted to be less than 1/1000 of the ingested RSV.

These results demonstrated that most of RSV was metabolized in the intestinal tract or excreted, and small amounts were absorbed and distributed in the body. Furthermore, colonization of Li01 facilitated and promoted the metabolism of RSV.

### 3.4. Resveratrol Metabolism in Conventional Mice and DSS-Induced Colitis Mice

We further investigated RSV metabolism in gut microbiota in either states of homeostasis or dysbiosis. Conventional mice and DSS-induced colitis mice were adopted as mouse models.

**Figure 4:**
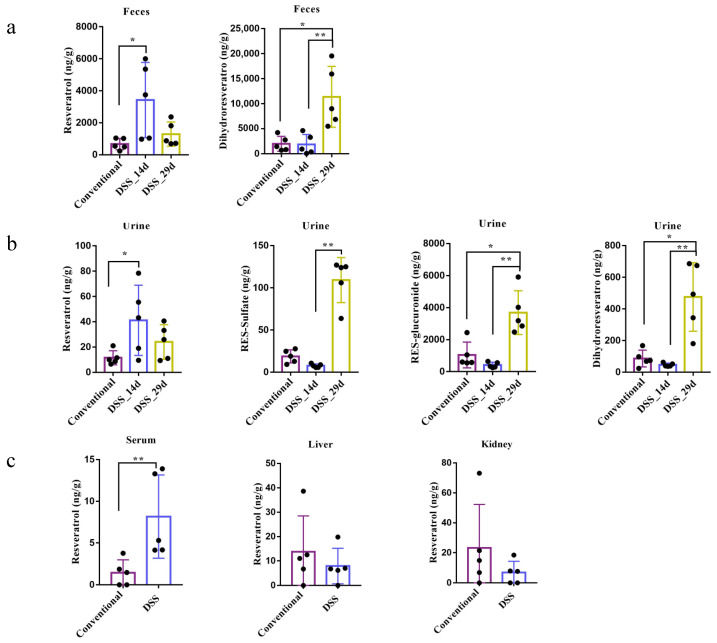
RSV metabolism and distribution in conventional mice and DSS-induced colitis mice after oral administration. RSV and its metabolites were quantified in feces (**a**), urine (**b**), serum, and tissues (**c**) of both conventional mice and DSS-induced mice. 14 d and 29 d present different duration of RSV intervention. Data are presented as mean ± SEM (*n* = 5). Significant difference was represented by * *p*  <  0.05, ** *p*  <  0.01, and analyzed by independent-samples *t*-test and one-way ANOVA with LSD’s post hoc test or nonparametric test (Mann–Whitney U-test and Kruskal–Wallis H test).

Since long-term RSV treatment may help to restore the gut microbiota, the metabolism of RSV can change over time. Thus, the RSV intervention on mice in the colitis model was divided into two stages: the first stage of treatment (14 d) and the later treatment (29 d). The result (Figure 4a) showed that in the conventional group, the concentration of RSV in feces is very low (below 1000 ng/g) while it significantly increased (averaged 6000 ng/g) in the DSS group (14 d), indicating that dysbiosis of gut microbiota may have impact on the metabolism of RSV. Long-term ingestion of RSV may promote the recovery of gut microbiota so that the level of RSV was reduced at 29 d. Besides, the level of DHR significantly increased among mice in the DSS group (29 d) compared to mice in the other two groups, which could be associated with the enhanced ratio of *Firmicutes/Bacteroidetes*. Some *Lactobaclillus* spp. may promote the metabolism of RSV into DHR. In addition, RSV-sulfate and RES-glucuronide were not detected in the fecal samples. One explanation is that gut microbiota in conventional and DSS mice may be able to deconjugate RES-sulfate and RES-glucuronide, while germ-free mice did not have gut microbiota to do this job. Besides, previous research indicated that conjugated metabolites of RSV were relatively stable in the presence of gut microbes [22]. RES-sulfate and RES-glucuronide usually form in the enterocyte and undergo the enterohepatic circulation. They may exit the cell via BRCP and MRP2 transporters on the apical membrane [25]. Taken together, it suggests gut microbiota play important roles in RSV metabolism.

RSV and its metabolites were also determined in the urine samples (Figure 4b). The concentration of RSV showed no significant difference among mice in the three groups. Interestingly, the levels of RES-sulfate, RES-glucuronide and DHR were all dramatically increased in mice in the DSS group (29 d) compared to mice in the conventional group or in the DSS group at the 14th day, indicating that large amount of RSV were metabolized and excreted after long-term modulation of the gut microbiota by RSV. Very low concentration of RSV was detected in serum, liver and kidney, which is similar to that in germ-free mice.

## 4. Conclusions

In conclusion, we demonstrated that gut microbiota composition plays an important role in controlling the metabolism of RSV. The probiotic strain Li01 promoted larger amounts of RSV metabolizing into DHR, RES-sulfate and RES-glucuronide. The level of DHR increased most significantly among the metabolites. The presence of gut microbiota stimulates the production of DHR at a higher level whereas the elimination of RES-sulfate and RES-glucuronide is promoted. Furthermore, we showed that long-term ingestion of RSV altered the gut microbiota of DSS-induced mice, and the ratio of *Firmicutes/Bacteroidetes* dramatically improved, which significantly boosted the metabolism of DHR and elevated the excreted level of RSV metabolites. In addition, gut microbiota did not greatly affect the distribution of RSV in the liver and kidney. Further investigation may be needed to fully understand the interactions among RSV, the gut microbiota, and its host.

## Figures and Tables

**Table 1:** List of resveratrol metabolites identified with LC-MS.

No.	Compounds	*m/z* [M-H]	MS/MS Fragment
1	RSV	227.0708	227.0702, 185.0790
2	RES-sulfate	307.027	307.0269, 185.0595
3	RES-glucuronide	403.1029	403.1029, 227.0703
4	DHR	229.0865	229.0837, 123.0442

## Data Availability

Not applicable.
